# A social cognitive theory of customer value co-creation behavior: evidence from healthcare

**DOI:** 10.1108/JHOM-02-2024-0074

**Published:** 2024-10-17

**Authors:** Md Moynul Hasan, Yu Chang, Weng Marc Lim, Abul Kalam, Amjad Shamim

**Affiliations:** Northwestern Polytechnical University, Xi'an, China; Sunway Business School, Sunway University, Petaling Jaya, Malaysia; School of Business, Law and Entrepreneurship, Swinburne University of Technology, Hawthorn, Australia; Faculty of Business, Design and Arts, Swinburne University of Technology – Sarawak Campus, Kuching, Malaysia; Curtin University Malaysia, Miri, Malaysia; Universiti Teknologi PETRONAS, Seri Iskandar, Malaysia

**Keywords:** Customer, Co-creation, Experience, Engagement, Self-efficacy, Value co-creation behavior

## Abstract

**Purpose:**

Customer value co-creation behavior is promising but undertheorized. To bridge this gap, this study examines the viability of a social cognitive theory positing that customers' value co-creation behavior is shaped by their co-creation experience, self-efficacy, and engagement.

**Design/methodology/approach:**

Using healthcare as a case, a stratified random sample comprising 600 patients from 40 hospitals across eight metropolitan cities in an emerging economy was acquired and analyzed using co-variance-based structural equation modeling (CB-SEM).

**Findings:**

Customers' co-creation experience has a positive impact on their co-creation self-efficacy, co-creation engagement, and value co-creation behavior. While co-creation self-efficacy and engagement have no direct influence on value co-creation behavior, they do serve as mediators between co-creation experience and value co-creation behavior, suggesting that when customers are provided with a co-creation experience, it enhances their co-creation self-efficacy and engagement, ultimately fostering value co-creation behavior.

**Originality/value:**

A theory of customer value co-creation behavior is established.

## 1. Introduction

Service is evolving to emphasize diverse, in-depth experiences that cater to customers' unique needs (Anderson *et al.*, 2018; Moorman *et al.*, 2024), enhancing their wellbeing (Akter *et al.*, 2022) and quality of life (Islam *et al.*, 2024). This marks a shift from the traditional provider-centric model to one that puts the customer in the spotlight and involves them in the process (McColl-Kennedy *et al.*, 2017a; Vogus *et al.*, 2020), promoting personalized experiences that meet individual customer requirements (Fisk *et al.*, 2018; Moorman *et al.*, 2024). While firm-customer collaborations enhance experience quality and outcomes, there is a notable gap in understanding how firms can effectively approach customers in value co-creation (George *et al.*, 2024; Nickel *et al.*, 2018). Indeed, creating optimal experiences is challenging (Nadeem *et al.*, 2021), but firms that excel in this area see increased profitability and loyalty (Ponsignon *et al.*, 2015).

As co-creators with contemporary resources and insights (Grönroos, 2011; Shamim *et al.*, 2016), customers should be treated as crucial stakeholders (Islam *et al.*, 2024; Vargo and Lusch, 2016). Since customers are experts in managing their own needs (Seiders *et al.*, 2015), their involvement as equal partners, makers, and shapers (Hau and Thuy, 2022; Janamian *et al.*, 2016) in co-creation is vital for fostering innovation and tailoring products and services to meet those needs (Gallan *et al*., 2019; Hussain *et al.*, 2021), thus holding immense potential for improved results and minimized complications (Kim, 2019; Nickel *et al.*, 2018). This engagement, combined with resources and skills, such as self-efficacy, can develop necessary competencies and result in desired outcomes (Frow *et al.*, 2019; Kim, 2019; Xie *et al.*, 2020). Firms are therefore focused on devising strategies to integrate these inputs, recognizing that value co-creation is essential in service design, development, and delivery (Grönroos and Gummerus, 2014; Hau *et al.*, 2017; Hau and Thuy, 2022; Moorman *et al.*, 2024). However, the debate about who truly drives value remains ongoing, marking this as an emerging research domain (Fusco *et al.*, 2023; Shamim *et al.*, 2016; Yen *et al.*, 2020).

Existing literature emphasizes the necessity of examining both the antecedents (McColl-Kennedy *et al.*, 2017a) and outcomes (Pham *et al.*, 2019) of co-creation to develop a holistic understanding. Yet, despite firms acknowledging the importance of co-creative experiences for competitive advantage and growth (Prahalad and Ramaswamy, 2004), there is a scarcity of empirical studies treating co-creation experience as a precursor to value co-creation (Nadeem *et al.*, 2021). Similarly, while research on co-creation and its outcomes is gaining traction in both academia and industry (Fusco *et al.*, 2023; Ranjan and Read, 2016), particularly within service sectors (McColl-Kennedy *et al.*, 2017a; Pham *et al.*, 2019; Xie *et al.*, 2020), a clear gap persists regarding the manifestations of value co-creation behavior. Given that these gaps continue to be called out by recent scholars (Fusco *et al.*, 2023; Hyder *et al.*, 2024; Vogus *et al.*, 2020), this study endeavors to answer this call and address these gaps. Specifically, this study posits that co-creation experience, characterized by personalization and relative advantage, is essential for stimulating positive outcomes, including co-creation self-efficacy, co-creation engagement, and value co-creation behavior (co-creation citizenship behavior and co-creation participation behavior).

Healthcare provides a suitable context for this study due to the inherent nature of patient involvement in the co-creation of value. Patients actively participate by communicating symptoms and conditions, aiding in diagnosis, sharing insights on treatment alternatives, and expressing preferences and comfort levels (Gallan *et al.*, 2019; Xie *et al.*, 2020). The holistic experience of consulting and co-creating with healthcare providers goes beyond recovery objectives (Islam *et al.*, 2024; Osei-Frimpong *et al.*, 2015). Engaged patients can offer innovative insights that enhance service performance and co-construct improved healthcare offerings, benefiting both patients and healthcare establishments (Jaakkola *et al.*, 2015; Vogus *et al.*, 2020). Collaborative value or “value-in-use” epitomizes customer engagement in the process, with customer experience being an indispensable component (Assiouras *et al.*, 2019; Grönroos and Voima, 2013; Yen *et al.*, 2020). Leveraging patient input in value co-creation promises mutual value and sustained collaborative efforts (Ciasullo *et al.*, 2022).

Modern marketing practices emphasize delivering unparalleled value through continuous customer engagement and resource sharing (Harrigan *et al.*, 2021; Kotler, 2017; Moorman *et al.*, 2024; Pansari and Kumar, 2017). Similarly, in healthcare, patient engagement involves sharing critical health information, emotional states, available resources, and preferences amid diverse treatment options (Gallan *et al.*, 2019). However, challenges arise as patients sometimes exhibit hesitancy in engaging in the co-creation process, and their inputs can be overlooked by healthcare professionals (McColl-Kennedy *et al.*, 2017b; Pekonen *et al.*, 2020). Despite these challenges, the intrinsic nature of healthcare services being directly related to individual wellbeing necessitates organic patient engagement (George *et al.*, 2024; Ramaswamy and Ozcan, 2016). Emphasizing patient engagement and their shared resources, such as self-efficacy, is crucial for successful treatment execution. Moreover, the Sustainable Development Goals (SDGs) emphasize accessible healthcare for all by 2030, highlighting the need for broad patient engagement in value co-creation. In line with this, the service-dominant (SD) logic advocates for customers as integral to co-creation (Vargo and Lusch, 2008, 2016). Embracing co-creation processes can foster customer feedback and reviews, potentially becoming a significant promotional asset for healthcare firms (Grönroos and Voima, 2013; Moorman *et al.*, 2024).

Social cognitive theory (SCT) guides this study by positing that behavior is influenced by the interaction of cognitive or personal factors, behavioral patterns, and environmental conditions (Bandura, 1986). SCT is particularly relevant for this study because it emphasizes the importance of self-efficacy and engagement (Bandura, 1977, 1997, 1998), which are critical components in the co-creation process. SCT involves observational learning and reinforcement learning (Bandura, 1986), both of which are implicitly represented in this study. Observational learning refers to individuals learning behaviors by observing others, which in the context of co-creation, can be seen when patients learn and adopt co-creation behaviors by observing and interacting with healthcare providers and other patients. Reinforcement learning pertains to how the consequences of an action influence the likelihood of that action being repeated. In co-creation, positive reinforcement through successful and satisfying co-creation experiences enhances patients' self-efficacy and engagement, encouraging continued participation in value co-creation activities. Therefore, by integrating SCT, this study highlights how co-creation experiences influence patients' confidence in their ability to co-create (self-efficacy) and their willingness to engage in the process (engagement). The study also considers the environmental factor—i.e. the healthcare setting—and how it interacts with personal factors to shape value co-creation behavior. This holistic approach ensures that the study comprehensively addresses the dynamics of co-creation, providing valuable insights into how healthcare providers can effectively foster patient involvement in co-creation activities. Therefore, while SCT encompasses observational and reinforcement learning, the scope of this study is primarily focused on the personal and behavioral aspects of SCT, specifically self-efficacy and engagement, in the context of co-creation experiences in healthcare. This focus allows for a detailed exploration of how these factors contribute to value co-creation behavior, filling a critical gap in existing research.

## 2. Literature review

### 2.1 Theoretical foundation: social cognitive theory (SCT)

Social cognitive theory (SCT) has been employed across diverse studies, including consumer psychology (Bandura, 1997), health promotion (Bandura, 1998, 2013), motivation (Schunk and DiBenedetto, 2020), and relational self (Andersen and Chen, 2002). The present study employs SCT as a lens to delineate and comprehend the relationships within value co-creation behavior. Utilizing SCT in this context remains a novel approach, especially in understanding the nexus of value co-creation behavior (AbdelAziz *et al.*, 2023; Zhao *et al.*, 2019), influenced by mediating factors such as self-efficacy and engagement (Bandura, 1977, 1997, 1998). SCT typically delineates how a confluence of cognitive or personal, behavioral, and environmental aspects shape behaviors (Bandura, 1986). Previous studies have often overlooked the individual aspect, particularly individuals' experiences, focusing instead on only personal and behavioral factors like self-efficacy and engagement as crucial catalysts for co-creation intention (AbdelAziz *et al.*, 2023; Zhao *et al.*, 2019). Schunk and DiBenedetto (2020) improvised these perspectives and suggested that, based on SCT, individuals develop a sense of self through interpersonal experiences and mutual interactions, which shape their identity, self-concept, and subsequent behavior. Additionally, Prahalad and Ramaswamy (2004) noted that value co-creation is considerably impossible without resource integration and engagement. Hence, by considering the co-creation experience as an inherent trait influencing self-efficacy and engagement, we can better understand the drivers and mechanisms that foster value co-creation behavior. Therefore, this study seeks to empirically explore these connections through SCT, aspiring to make a noteworthy contribution to the literature on value co-creation behavior in healthcare services.

### 2.2 Conceptual foundation: value co-creation behavior

Patient participation in co-creation is gaining momentum in healthcare service research, ensuring positive outcomes in the healthcare journey, such as improved quality of life and wellbeing (Anderson *et al.*, 2018; Islam *et al.*, 2024; McColl-Kennedy *et al.*, 2017b). Value co-creation behavior is a multifaceted construct (Yen *et al.*, 2020) that manifests in two primary ways: co-creation participation behavior and co-creation citizenship behavior (Yi and Gong, 2013). Co-creation participation behavior involves customers in the design and delivery of services (Yen *et al.*, 2020), representing a foundational role in value co-creation (Yi and Gong, 2013). Activities such as information seeking, information sharing, responsible behavior, and personal interactions are integral to co-creation participation behavior (Hau *et al.*, 2017). Conversely, co-creation citizenship behaviors are customer-led initiatives that offer added value to firms through feedback, advocacy, helping, and tolerance (Yen *et al.*, 2020; Yi and Gong, 2013). Active engagement is crucial for successful collaboration in the co-creation process (Prahalad and Ramaswamy, 2004; Vargo and Lusch, 2016), and resource integration is paramount. Patients, being skilled and knowledgeable about their health and complications (Seiders *et al.*, 2015), make the co-creation process successful, ensuring quality of life and wellbeing (Islam *et al.*, 2024; McColl-Kennedy *et al.*, 2017b). Embracing feedback and recommendations from customers is pivotal in enhancing service offerings, promoting word of mouth, and assisting other potential users (Burnham *et al.*, 2020; Moorman *et al.*, 2024).

## 3. Hypothesis development

### 3.1 Co-creation experience and value co-creation behavior

Grounded in SCT, customer experience can shape behavior (Schunk and DiBenedetto, 2020). Customer experience emanates from direct or indirect interactions between customers and firms (Homburg *et al.*, 2017; Hussain *et al.*, 2021). Incorporating customers is paramount for co-creating value in service delivery (Edvardsson *et al.*, 2011; Prahalad and Ramaswamy, 2004), with experience being a pivotal component in enhancing the process of value co-creation (Grönroos, 2011). Co-creation experience necessitates genuine engagement within the service co-design paradigm (Yen *et al.*, 2020). Such experiences, whether positive or negative, mold customer cognition, catalyzing behaviors (Heinonen and Strandvik, 2015) such as value co-creation behavior, a critical process wherein customers and service providers collaborate to generate value through mutual interactions and resource exchange (Vargo and Lusch, 2016). This mutual interaction can manifest as customers playing active roles in service design or suggesting enhancements for service outcomes.

Prevailing research indicates that a customer's co-creation experience, characterized by personalization and relative advantage, encourages deeper involvement in activities, fostering future participation in value co-creation (Chatterjee *et al.*, 2022). Essentially, personalization embodies the sentiments customers derive from tailored services (Neuhofer *et al.*, 2015). In marketing, personalization correlates with the singularity of service outcomes, anchored by individual-centric factors (Chatterjee *et al.*, 2022; Karpen *et al.*, 2012). Consequently, custom services or personalized counsel could stimulate desired customer behaviors as they are vital for catering to the distinctive needs and preferences of customers (Chandra *et al.*, 2022; Vogus *et al.*, 2020). Whereas, relative advantage pertains to the facet of a customer's experience drawn from comparing varied service encounters (Chatterjee *et al.*, 2022; Wei *et al.*, 2015), enabling customer awareness of the discerned benefits of engaging in co-creation versus traditional services (Vogus *et al.*, 2020), which in turn fosters engagement, loyalty, and evangelistic behavior (Harrigan *et al.*, 2021; Leckie *et al.*, 2018; Ponsignon *et al.*, 2015). Both personalization and relative advantage, as first-order constructs, form the foundational elements of co-creation experience, as a second-order construct, enhancing co-creation effectiveness and future co-creation engagements (Chatterjee *et al.*, 2022). Scholars have employed a similar second-order construct of smart customer experience to scrutinize resultant behaviors (Roy *et al.*, 2017). However, the linkage between co-creation experience and value co-creation behavior has not been extensively explored. This study endeavors to bridge this gap. Hence, the following hypothesis is postulated:

H1. Co-creation experience has a positive influence on value co-creation behavior.

### 3.2 Co-creation experience and co-creation self-efficacy

Rooted in SCT, experience serves as a robust predictor of self-efficacy (Bandura, 1986), which in turn influences subsequent behaviors (Bandura, 1977; Schunk and DiBenedetto, 2020). The duration of a customer's association with a firm is significantly influenced by the experiences garnered from that firm (Kumar Roy *et al.*, 2014). Cognitive and learning processes arising from behavioral activities, active participation, spontaneity, and integrative roles can foster an enriched sense of experience (Heinonen and Strandvik, 2015). Moreover, service experience has been shown to bolster self-efficacy, which cascades into future customer behaviors (Thakur, 2018). Equipping customers through valuable experiences and fostering their capacities can enhance their self-efficacy, which is beneficial in value co-creation (Alves *et al.*, 2016). Empowering customers, coupled with a rewarding co-creation experience, can therefore influence their self-efficacy, ultimately guiding them toward value co-creation behavior. As customers gain confidence in their ability to contribute effectively through positive co-creation experiences, their self-efficacy is strengthened, promoting a greater willingness to engage in co-creation activities. Consequently, the study postulates:

H2. Co-creation experience has a positive influence on co-creation self-efficacy.

### 3.3 Co-creation self-efficacy and value co-creation behavior

SCT underscores that individuals with heightened self-efficacy levels exhibit increased confidence and motivation, leading them to actively engage in interactions and secure improved outcomes (Bandura, 1997; Schunk and DiBenedetto, 2020). Self-efficacy not only propels future behavior (Bandura, 1986) but also catalyzes proactive behaviors in specific contexts (Bartle and Harvey, 2017). Essentially, self-efficacy delineates the potential actions that individuals, equipped with specific abilities and skills, can undertake (Zhao *et al.*, 2019). Collaborative engagements foster dialogue, knowledge exchange, new learning, and the creation of novel insights, laying the groundwork for continuous value co-creation (Alves and Wagner Mainardes, 2017; Ciasullo *et al.*, 2022). The impetus for individuals to engage in value co-creation is contingent upon key determinants, with self-efficacy being paramount (Zhao *et al.*, 2019). Elevated levels of self-efficacy have been linked to a heightened interest in co-creation processes, translating into valuable contributions (Zhang *et al.*, 2019). Prior research has highlighted the positive relationship between self-efficacy, knowledge sharing, and the willingness to co-create value (Alves *et al.*, 2016), influence behavior (Bravo *et al.*, 2020), and engage in co-creation activities (Alves and Wagner Mainardes, 2017). However, the relationship between co-creation self-efficacy and value co-creation behavior remains sparsely explored in extant literature. To address this gap, the study postulates:

H3. Co-creation self-efficacy has a positive influence on value co-creation behavior.

### 3.4 Co-creation experience and co-creation engagement

Drawing from SCT, an individual's experience is pivotal, potentially affecting their interests, reinforcements, and expectancies when exhibiting behavior (AbdelAziz *et al.*, 2023; Bandura, 1986; Schunk and DiBenedetto, 2020). In the context of co-creation, customers' engagement in the service design process is crucial (Yen *et al.*, 2020). Previous studies affirm that positive brand experiences enhance brand affinity, leading to increased engagement (Prentice *et al.*, 2019). Additionally, research highlights the role of experience in influencing value co-creation (Lee, 2019). Therefore, it is imperative for service firms to ensure a valuable co-creation experience, as it is paramount for enhancing engagement (Brodie *et al.*, 2019; Hollebeek *et al.*, 2019). Positive co-creation experiences can heighten engagement, fostering behaviors such as referrals, influencing potential customers, and providing valuable feedback (Hussain *et al.*, 2021). This, in turn, strengthens co-creation efforts (Wu and Gao, 2019) and encourages value co-creation behavior (Nadeem *et al.*, 2021). Despite these findings, the direct link between co-creation experience and engagement remains relatively uncharted. Thus, the following hypothesis is proposed:

H4. Co-creation experience has a positive influence on co-creation engagement.

### 3.5 Co-creation engagement and value co-creation behavior

SCT underscores that interpersonal interactions foster customer behavior, emphasizing the importance of engagement in shaping these interactions (Schunk and DiBenedetto, 2020). Engagement is central to co-creating value, facilitating a dynamic and reciprocal process between customers and firms (Brodie *et al.*, 2019). When customers are engaged, they are more likely to actively participate in co-creation activities, which leads to optimal outcomes (Vargo and Lusch, 2016). Engaged customers tend to resonate with services both during consumption and post-consumption, enhancing their overall experience and satisfaction (Karpen and Conduit, 2020). Furthermore, highly engaged customers often exhibit enhanced participatory behavior, which positively influences their involvement in the co-creation process (Hollebeek *et al.*, 2019; Nadeem *et al.*, 2021). This engagement fosters positive attitudes toward firms, influencing loyalty and advocacy (Vivek *et al.*, 2012). The strong connection between engagement and value co-creation is thus seen as instrumental in marketing paradigms (Vivek *et al.*, 2012; Yen *et al.*, 2020). Engagement and co-creation are interrelated concepts in service research, both pivotal to value creation (Jaakkola and Alexander, 2014). Research has shown that engagement impacts value co-creation intentions, identifying elements that enhance co-creation with brands (AbdelAziz *et al.*, 2023; France *et al.*, 2015). Despite the evident role of engagement in catalyzing value co-creation behavior, the specific connection between engagement and value co-creation behavior has not been exhaustively explored. Therefore, the subsequent hypothesis is introduced:

H5. Co-creation engagement has a positive influence on value co-creation behavior.

### 3.6 Co-creation self-efficacy and co-creation engagement

As per SCT, self-efficacy facilitates engagement, enhancing overall outcomes (Bandura, 1997; Schunk and DiBenedetto, 2020). Self-efficacy pertains to an individual's perception of their abilities and skills (Zhao *et al.*, 2019), signifying their capability to participate in co-creation (Zhang *et al.*, 2019). Existing research underscores the importance of self-efficacy as a foundational requirement for behaviors such as engagement (Bravo *et al.*, 2020). When individuals possess a high level of self-efficacy, they are more motivated to involve themselves deeply in co-creation activities (Alves and Wagner Mainardes, 2017). This motivation significantly impacts their engagement in the co-creation process, especially when customers share their knowledge and expertise with firms (Zhang *et al.*, 2019). Those with a strong sense of capability often feel more responsible and inclined to contribute positively to co-creation efforts (Zhang *et al.*, 2019). Multiple studies have asserted that self-efficacy functions as a crucial catalyst affecting engagement (Bravo *et al.*, 2020). Drawing upon SCT, self-efficacy has been evidenced to influence customer engagement, which is pivotal in augmenting co-creation (AbdelAziz *et al.*, 2023). Despite its importance, the relationship between co-creation self-efficacy and engagement remains relatively underinvestigated. Consequently, the following hypothesis is posited:

H6. Co-creation self-efficacy has a positive influence on co-creation engagement.

Customer engagement in co-creation and co-creation self-efficacy play significant roles as mediators in fostering value co-creation behavior. Self-efficacy, derived from one's experiences and interactions, motivates behavior (Schunk and DiBenedetto, 2020), and thus, in healthcare settings, it is posited that patients with high self-efficacy are more likely to engage actively in their care and co-creation behaviors. Noteworthily, Bartle and Harvey (2017) noted that healthcare behavior is a product of self-efficacy, particularly when self-efficacy is guided by experience. Furthermore, patient engagement intensifies active participation in co-creation and collaboration between patients and healthcare professionals in treatment plans, execution, and shared decision-making throughout the healthcare journey. Previous studies, such as Yen *et al.* (2020), underscore that engagement enhances the likelihood of positive value co-creation behavior and serves as a mediator between innovativeness and value co-creation behavior, wherein patients who possess self-efficacy from direct co-creation experience are likely to engage actively with healthcare professionals, developing a sense of empowerment and ownership over their healthcare journey. This increased ability and motivation to contribute meaningfully to value co-creation ultimately leads to improved health outcomes and patient satisfaction. Based on this discussion and the aforementioned literature on proposed direct relationships, the following mediating relationships are hypothesized:

H7. Co-creation self-efficacy mediates the relationship between co-creation experience and value co-creation behavior.

H8. Co-creation engagement mediates the relationship between co-creation experience and value co-creation behavior.

H9. Co-creation self-efficacy mediates the relationship between co-creation experience and co-creation engagement.

**Figure 1:**
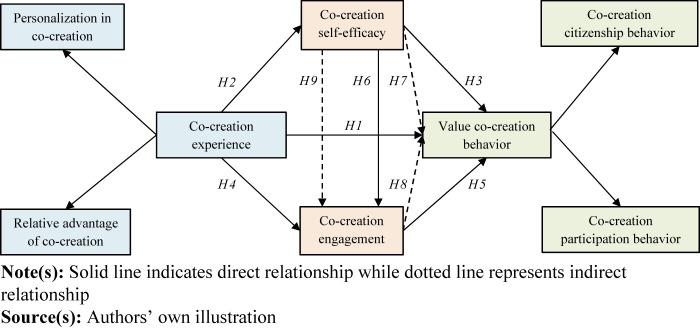
Conceptual model

The conceptual model illustrating and summarizing the hypothesized direct and mediating relationships is presented in Figure 1.

## 4. Methodology

### 4.1 Instrumentation

A survey was conducted using a questionnaire, which consists of items adapted from extant literature. Co-creation experience is a second-order construct comprising personalization and relative advantage as first-order constructs. For personalization, items were adapted from Neuhofer *et al.* (2015) while relative advantage items were adapted from Lowe and Alpert (2015) and Wünderlich *et al.* (2015). Self-efficacy, engagement, and value co-creation behavior were adapted based on items from McKee *et al*. (2006), Vivek *et al.* (2012), and Yi and Gong (2013), respectively. The initial set consists of 35 items, but only 26 items with factor loadings above 0.70 (or above 0.60 and deemed theoretically important)—and thus demonstrating convergent validity—were retained (Hair *et al*., 2010). Given that the context of the study is healthcare, the questions were tailored for patients of healthcare institutions.

As an initial filter, respondents were queried about their recent interactions with hospitals, specifically inquiring if they had visited any in the recent past for healthcare services. If the response was affirmative (yes), respondents answered a series of general questions to gauge their experiences. Subsequent sections required participants to indicate their level of agreement or disagreement with specific statements (items) using a 7-point Likert scale, where 1 denoted “strongly disagree” and 7 signified “strongly agree”.

To cater to the linguistic preferences of respondents, survey questions were presented in both English and Bengali, with forward and backward translation conducted and agreed upon by the main author and another linguistic expert—both had a good command of both languages (Siraji and Haque, 2022). The questionnaire underwent a *pretest* with two professors with expertise in co-creation to ensure *content validity*, and a subsequent *pilot study* with 65 participants was conducted to ensure *face validity* (Lim, 2024). Minor adjustments for clarity were made based on the feedback from the pretest and pilot study before the questionnaire was administered in the main study.

### 4.2 Data collection

The primary target group comprised individuals who had visited hospitals in major metropolitan cities in an emerging economy, Bangladesh, specifically Barishal, Chattogram, Dhaka, Khulna, Mymensingh, Rajshahi, Rangpur, and Sylhet. The focus on metropolitan cities was intentional, given that specialized healthcare services and professionals predominantly reside in these urban centers, drawing patients nationwide.

For our data collection, we administered questionnaires to 1,200 individuals across 40 hospitals in the aforementioned cities. From this effort, we received 609 responses, representing a response rate of 50.75%. After data cleaning (excluding incomplete and outlier data), 600 of these responses were retained for further analysis. Eligibility criteria for participants included being a minimum age of 18 and having visited a hospital for healthcare services within the preceding three months. This timeframe was chosen to ensure participants could accurately recall their recent service experiences (Hussain *et al.*, 2021; Xie *et al.*, 2020), as recall accuracy diminishes over time, and a three-month window strikes a balance between recency and participant availability, thereby enhancing the validity of the responses and the overall quality of the data collected in an accessible, pragmatic manner (Lim, 2024).

Our sampling strategy employed stratified random sampling, favored for its simplicity, representativeness, and frequent application in healthcare research (Dahl *et al.*, 2021; Kang *et al.*, 2021). Initially, the metropolitan areas were divided into distinct strata. Following this, patient visitation records from the hospitals were examined, and before distributing our survey, we secured approval from the administrative bodies of each hospital. Potential participants were then contacted via phone to secure their consent, with questionnaires subsequently being emailed.

The questionnaire solicited demographic information and insights into participants' co-creation experiences within healthcare. A breakdown of the demographics reveals that 83.2% of respondents were male, and the age bracket of 27–53 years encompassed 91.2% of the sample (Table 1). In terms of occupation, the majority were either private job holders (37.1%) or educators (31.1%). Frequency of hospital visits varied, with 30.1% having frequented healthcare institutions over two to three years, 25.7% for less than two years, and 23.8% over a span above seven years. In terms of institution type, 63.0% of respondents had visited private, 24.9% governmental, and 5.8% autonomous healthcare institutions.

### 4.3 Data analysis

To analyze the data, this study employed co-variance-based structural equation modeling (CB-SEM) using AMOS *v.*24. At its core, CB-SEM encompasses a variety of statistical models (measurement, structural) designed to articulate and test theoretical associations (Astrachan *et al.*, 2014). One of the inherent strengths of CB-SEM is its capability to establish the reliability and validity of data, while providing a comprehensive assessment of the relationships between constructs (Anderson and Gerbing, 1988). This method also facilitates a rigorous examination of key model fit indicators, ensuring the data's alignment with the proposed conceptual model. Given the objectives of this study, which include assessing model fit, verifying the reliability and validity of constructs, and analyzing the relationships between variables, CB-SEM was deemed as an appropriate analytical tool. In addition to the primary analysis, a bootstrap analysis was executed using AMOS *v.*24, enabling a more detailed evaluation of the mediating effects present within the model.

## 5. Results

### 5.1 Measurement model

To validate our measurement model, we first ascertained its reliability or internal consistency, convergent validity, discriminant validity, and potential multicollinearity (Tables 2 and 3). Subsequent evaluations included common method bias, normality test, skewness and kurtosis assessment, and identification of outliers through the Mahalanobis distance.

To begin, the *reliability* metrics affirm the *internal consistency* of our scales: all Cronbach's alpha and composite reliability (CR) values surpassed the recommended threshold of 0.70 (Fornell and Larcker, 1981). Next, the standardized factor loadings and average variance extracted (AVE) for each construct were both well within the acceptable range, with all factor loadings exceeding 0.70 (or above 0.60 and deemed theoretically important) and AVE values surpassing 0.50, thus reflecting *convergent validity* (Hair *et al*., 2010). Furthermore, the square roots of the AVEs surpass their associated correlation values with other constructs, thereby reflecting *discriminant validity* (Hair *et al*., 2010). Furthermore, in terms of potential *multicollinearity*, the measurement model demonstrated good integrity, as the variance inflation factors (VIF) remained within the widely accepted threshold of 10 (as well as the more stringent threshold of 3.3), and the tolerance values too were comfortably ensconced within their prescribed limits. Focusing on the overall fit of the measurement model, we found that most of the indices met or exceeded their benchmarks. Notably, the goodness-of-fit metrics such as relative chi-square (*χ^2^*/df), goodness-of-fit index (GFI), adjusted goodness of fit index (AGFI), comparative fit index (CFI), incremental fit index (IFI), normed-fit index (NFI), Tucker and Lewis index (TLI), and root mean square error of approximation (RMSEA) registered values of 1.955 (<3), 0.934 (≥0.90), 0.918 (≥0.90), 0.970 (≥0.90), 0.970 (≥0.90), 0.940 (≥0.90), 0.966 (≥0.90), and 0.040 (<0.08), respectively (Hair *et al*., 2010). Moreover, we observed that the maximum shared variance (MSV) was outperformed by the AVE. The MaxR (H) values also exceeded the CR values. Similarly, the second-order constructs for co-creation experience and value co-creation behavior also demonstrated satisfactory fit, meeting the established criteria for adequate goodness-of-fit, with metrics like *χ^2^*/df = 2.659 (<3), GFI = 0.939 (≥0.90), AGFI = 0.920 (≥0.90), CFI = 0.969 (≥0.90), IFI = 0.969 (≥0.90), NFI = 0.951 (≥0.90), TLI = 0.964 (≥0.90), and RMSEA = 0.053 (<0.08) aligning with their respective benchmarks (Hair *et al*., 2010).

Considering potential *common method bias*—a concern when gathering data from a singular source at a particular time—it is worth noting that such biases, although undesirable, can be inherent in survey-based studies. Drawing inspiration from the procedural and statistical approaches delineated by Hosen *et al*. (2021) and Lim (2024), several steps were taken to mitigate this bias. These measures included leveraging measuring items from prior research, conducting a pretest with experts and a pilot study using intended respondent samples, employing a question randomization strategy, and designing the questionnaire with distinct scales to foster objective thinking. Nevertheless, to quantitatively assess the presence of common method bias, we utilized the single-factor Harman test. Results were promising, with the primary component accounting for only 35.34% of the variance—well below the 50% criterion posited by Hair *et al*. (2010). Similarly, *skewness* and *kurtosis* metrics comfortably nestled within the range of −2 to +2 (Tabachnick and Fidell, 2001) and −7 to +7 (Byrne, 2013) respectively, signifying *normal* distribution, which is further reaffirmed by the Mahalanobis distance, which showed no indication of significant *outliers*. Collectively, these metrics underscore that our study's measurement model boasts a good fit, further substantiating the soundness of our research approach and methodology.

### 5.2 Structural model

Utilizing CB-SEM in AMOS *v*.24, we evaluated the structural model. Our structural model's fit indices largely conformed to the standards: *χ^2^*/df = 1.986 (<3), GFI = 0.932 (≥0.90), AGFI = 0.917 (≥0.90), CFI = 0.968 (≥0.90), IFI = 0.968 (≥0.90), NFI = 0.938 (≥0.90), TLI = 0.964 (≥0.90), and RMSEA = 0.041 (<0.08) (Hair *et al*., 2010).

**Figure 2:**
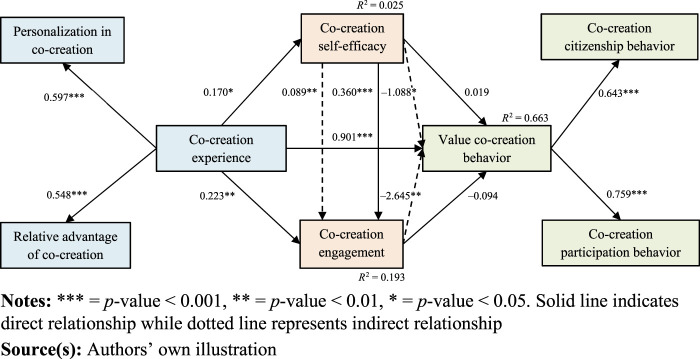
Structural model

Following that, we assess the hypothesized relationships (Figure 2, Tables 4 and 5). We found a positive and significant relationship between co-creation experience and both value co-creation behavior (*β* = 0.901, b = 1.132, S.E. = 0.180, C.R. = 6.300, *p* = 0.000 < 0.001) and co-creation self-efficacy (*β* = 0.170, b = 0.237, S.E. = 0.095, C.R. = 2.482, *p* = 0.013 < 0.05), supporting H1 and H2 respectively. Conversely, while the relationship between co-creation self-efficacy and value co-creation behavior was positive, it did not achieve statistical significance (*β* = 0.019, b = 0.017, S.E. = 0.060, C.R. = 0.291, *p* = 0.771 > 0.05), rendering H3 unsupported. Similarly, though co-creation experience positively and significantly influenced co-creation engagement (*β* = 0.223, b = 0.252, S.E. = 0.080, C.R. = 3.165, *p* = 0.002 < 0.01) in support of H4, the subsequent link between co-creation engagement and value co-creation behavior proved to be both negative and statistically insignificant (*β* = −0.094, b = −0.104, S.E. = 0.084, C.R. = -1.247, *p* = 0.212 > 0.05), leading to the rejection of H5. Nevertheless, co-creation self-efficacy was found to positively and significantly impact co-creation engagement (*β* = 0.360, b = 0.292, S.E. = 0.042, C.R. = 7.030, *p* = 0.000 < 0.001), supporting H6.

Finally, our study probed the potential mediating roles of co-creation self-efficacy and engagement between co-creation experience and value co-creation behavior. A significant indirect effect was observed for co-creation experience on value co-creation behavior via co-creation self-efficacy (*β* = −1.088, CI = −4.882, −0.008 *p* = 0.027 < 0.05) and engagement (*β* = −2.645, CI = −13.904, −0.040, *p* = 0.004 < 0.01), validating H7 and H8 respectively. Moreover, the indirect effect of co-creation experience on co-creation engagement through co-creation self-efficacy was also significant (*β* = 0.089, CI = 0.026, 0.173, *p* = 0.003 < 0.01), substantiating H9. In the presence of these mediators, the direct effect of co-creation experience on value co-creation behavior remained significant (*β* = 0.901, C.R = 6.300, *p* = 0.000 < 0.001). This indicates that both co-creation self-efficacy and engagement partially mediate the relationship between co-creation experience and value co-creation behavior, with co-creation self-efficacy also mediating the link between co-creation experience and engagement.

## 6. Discussion and conclusion

### 6.1 Evolution from service delivery to co-creation

The SD logic paradigm has reshaped business understanding by shifting the focus from merely delivering products and services to facilitating value co-creation. According to the SD logic paradigm, value is consistently co-created in collaboration with customers (Vargo and Lusch, 2016). This perspective clarifies the roles of actors within the service ecosystem and emphasizes the pivotal role of customers in realizing value through value-in-use. Noteworthily, the transition from a traditional service delivery model to one that prioritizes value co-creation heralds a paradigm shift in how firms approach and interact with customers. At the heart of this transformation is the recognition that value is not a static construct delivered to passive recipients. Instead, value becomes dynamic, emerging from the synergistic collaboration between providers and customers. This idea challenges the erstwhile “factory model” of service delivery, which viewed customers as mere end-users. In contrast, the SD logic paradigm champions customers as co-architects of value, placing them at the center of business operations.

From a theoretical standpoint, this shift necessitates a reevaluation of many established service theories. Whereas earlier theories might have overemphasized the roles of service providers in shaping outcomes, the SD logic paradigm necessitates an exploration of the mutual, reciprocal dynamics between providers and customers. This could lead to new conceptual frameworks that more holistically capture the nature of value creation, moving beyond linear cause-effect paradigms to embrace more cyclical, iterative models of value co-creation. A critical aspect of this shift is the reorientation from return on investment (ROI) to return on value (ROV). While ROI traditionally focuses on the financial gains relative to the cost of investment, ROV emphasizes the overall value generated, including intangible benefits such as customer satisfaction, loyalty, and long-term engagement. This broader perspective on value recognizes that the benefits of co-creation extend beyond immediate financial returns to encompass enhanced customer relationships and sustained business growth.

For practitioners, the SD logic paradigm offers both challenges and opportunities, demanding a reimagining of business operations to create platforms and environments conducive to customer participation. Traditional customer service metrics, which might have prioritized efficiency or speed, should now be complemented with measures assessing co-creation quality, customer empowerment, and collaborative value realization. Firms must acknowledge and accommodate the shift from ROI to ROV by fostering ongoing customer dialogues and investing in training and resources to ensure that front-line staff are equipped to collaborate effectively with customers. Feedback loops become crucial in this co-creation model, where customer feedback is not just a post-experience evaluation but an ongoing dialogue that continuously shapes and refines the service offering. This holistic approach emphasizes long-term engagement and satisfaction over immediate financial returns, recognizing that value is co-created with customers and includes both tangible and intangible outcomes. Consequently, firms are nudged to transition from a transactional mindset to one rooted in partnership and collaboration.

For healthcare providers, this means creating patient-centered care models where patients actively participate in their treatment plans. For example, hospitals could implement collaborative platforms where patients can share feedback on their care, leading to tailored treatments that improve patient outcomes and satisfaction. Policymakers can support this shift by encouraging the adoption of health IT systems that facilitate patient-provider communication and by creating regulations that incentivize patient involvement in healthcare decisions. Turning to public services, agencies can engage citizens in co-creating solutions to community problems, enhancing the effectiveness and acceptance of public policies. This approach not only contributes to enhancing trust but also ensuring that services are more responsive to the needs of the community.

### 6.2 Dynamics of co-creation experience and value co-creation behavior

The significance of co-creation experience in determining value co-creation behavior emerges as a salient theme from our findings, wherein experience transcends mere transactional interactions, encompassing the holistic journey a customer undergoes, from their initial expectations and the process of co-creation to the eventual outcomes they perceive. This experiential journey, imbued with emotion, cognition, and action, molds the very fabric of value co-creation behavior. When facilitated correctly, it transforms a passive recipient into an active participant, someone deeply invested in the value they co-create. Such active participation is not an end in itself but acts as a springboard, driving customers to become advocates, sharing their positive encounters and endorsing the service, enhancing its credibility and reach.

The SD logic asserts that co-creation experiences enhance emotional attachment, customer brand engagement, and evangelistic behavior (Harrigan *et al.*, 2021; Hussain *et al.*, 2021). This study demonstrates that when customers are allowed to co-create their healthcare services and are offered relatively better healthcare services, they feel motivated to actively participate in their healthcare journey, which deepens their connection with the service, prompting them to share positive feedback and recommendations. These findings highlight that the significant relationship observed between co-creation experience and value co-creation behavior is consistent with extant theoretical understanding, aligning with prior studies highlighting the significance of customer experience in magnifying value co-creation (Chatterjee *et al.*, 2022; Wu and Gao, 2019; Zhao *et al.*, 2019). When customers actively participate in co-creation, it deepens their connection with the service, prompting them to share positive feedback and recommendations, which corroborates findings from earlier studies like Ponsignon *et al.* (2015).

From a theoretical perspective, the association between co-creation experience and value co-creation behavior extends and reinforces existing theoretical understanding. This underscores the pivotal role of experience as not just an episodic touchpoint but as an ongoing narrative that shapes subsequent behaviors. The findings also prompt scholars to delve deeper into the nuances of experience. What constitutes a positive co-creation experience? How do contextual factors influence these experiences? And how do varying experiences influence the behavioral outcomes in the co-creation paradigm? The exploration of these questions could pave the way for a better understanding of the co-creation dynamics, moving beyond monolithic models to ones that embrace the multifaceted nature of customer experiences.

For practice, understanding the centrality of co-creation experience becomes imperative. This necessitates an overhaul of customer touchpoints, ensuring that each interaction is designed to foster a positive co-creation experience. Customer training, feedback mechanisms, and even technological platforms should be geared toward facilitating seamless, rewarding co-creation journeys. Moreover, firms might benefit from developing mechanisms to capture, analyze, and act upon experiential feedback in real-time, ensuring that they remain responsive and adaptive to the evolving needs of their co-creating customers. As customers become more entrenched in their co-creation roles, their experiences will serve as the lighthouse, guiding firms in their quest to realize optimal value.

For healthcare providers, integrating the principles of value co-creation into patient care can transform the healthcare experience. Providers can create patient-centered care models where patients actively participate in their treatment plans, thereby improving adherence and outcomes. For example, hospitals can implement digital platforms where patients and healthcare professionals collaborate on care plans, share real-time feedback, and adjust treatments based on patient input. Such platforms can enhance patient engagement and satisfaction, ultimately leading to better health outcomes.

For policymakers, the focus should be on creating supportive environments for value co-creation. Policymakers play a crucial role in facilitating these changes by promoting policies that support patient-provider collaboration. They can encourage the adoption of health IT systems that facilitate real-time communication and co-creation of care plans. Additionally, policies can incentivize healthcare providers to adopt value co-creation practices by linking reimbursements to patient engagement and satisfaction metrics. Accordingly, policymakers can help drive systemic improvements in healthcare quality and efficiency by fostering an environment that supports active patient involvement.

### 6.3 Dynamics of self-efficacy in co-creation

The study emphasizes the interplay between co-creation experiences and individuals' self-efficacy beliefs. Drawing inspiration from Bandura's (1986) SCT, it is evident that co-creation experiences can significantly mold a customer's confidence in their abilities to co-create. Positive experiences can act as catalysts, empowering customers to see themselves not as passive recipients but as vital collaborators. Following SCT perspectives (Andersen and Chen, 2002; Schunk and DiBenedetto, 2020), this study demonstrates that when customers are allowed to co-create their healthcare services and are offered relatively better personalized healthcare services, their co-creation self-efficacy and health maintenance capability are enhanced.

However, our findings introduce another noteworthy layer to this discourse. While heightened self-efficacy is expected to engender proactive behaviors, the translation is not as straightforward. The divergence between confidence and action highlights the complexities of value co-creation, underscoring that mere belief in one's capabilities might not be the sole determinant of co-creation behaviors. Instead, the intermeshing of individual beliefs and the broader context in which co-creation occurs dictates the outcomes. This complexity was evident in the study, where the direct effect of co-creation self-efficacy on value co-creation behavior was not significant. It was only when co-creation experience and co-creation self-efficacy were jointly considered that co-creation self-efficacy significantly facilitated the relationship between co-creation experience and value co-creation behavior. This can be logically explained by the fact that individuals may participate in the behavior without assessing their self-efficacy initially. As they gain experience, they realize the importance of self-efficacy, which then activates its influence in mediating the relationship between co-creation experience and value co-creation behavior. However, the influence was found to be negative, suggesting that individuals may recognize that the required co-creation self-efficacy is much higher than anticipated, thereby weakening the effect of co-creation experience on value co-creation behavior when mediated by co-creation self-efficacy. This finding is in line with Bartle and Harvey (2017), who claimed that vicarious experience motivates mothers' self-efficacy in breastfeeding behavior but also noted that self-efficacy alone is not sufficient to render mothers' breastfeeding intention.

The observed dynamics between co-creation experience, self-efficacy, and behavior offer rich avenues for theoretical exploration. While SCT provides a foundational understanding of how experiences shape self-beliefs, our findings suggest the need for extensions or complementary theories that consider the broader co-creation context. This raises intriguing questions for scholars: How do environmental factors, organizational cues, or even interpersonal dynamics shape the relationship between self-efficacy and co-creation behavior? Are there threshold levels of self-efficacy beyond which its influence on behavior diminishes? The subtle discrepancies between our findings and traditional SCT predictions emphasize the multifaceted nature of co-creation, warranting deeper, context-specific theoretical explorations.

For firms looking to harness the power of co-creation, understanding the pecularities of self-efficacy becomes paramount. It is not enough to craft positive co-creation experiences; there is a need to ensure these experiences are embedded within a context that facilitates the translation of heightened self-efficacy into tangible co-creation behaviors. This could involve refining communication strategies, ensuring clarity in roles during co-creation, providing the necessary tools or platforms for collaboration, and creating an organizational culture that genuinely values and responds to customer inputs. Firms should also consider interventions, such as training or workshops, that not only enhance self-efficacy but also equip customers with the knowledge and skills to actualize their co-creation potential.

For healthcare providers, enhancing patient self-efficacy is crucial for effective co-creation of care. Providers can develop patient education programs that build confidence in managing health conditions. For instance, chronic disease management programs can include workshops that teach patients how to monitor their symptoms, use medical devices, and communicate effectively with healthcare professionals. Such programs should be designed to provide positive co-creation experiences, thereby boosting patient self-efficacy and encouraging proactive health behaviors. Additionally, healthcare providers can implement support groups where patients share experiences and strategies, further enhancing their self-efficacy through peer learning.

Policymakers can support these initiatives by promoting policies that incentivize healthcare providers to focus on patient self-efficacy and co-creation. Policies could mandate the inclusion of self-management education in chronic disease management plans or provide funding for the development of digital health tools that facilitate patient-provider collaboration. Furthermore, policymakers can encourage the adoption of standardized measures for assessing patient self-efficacy and the impact of co-creation practices on health outcomes. Hence, policymakers can help drive systemic improvements in healthcare quality and efficiency by fostering an environment that supports active patient involvement.

### 6.4 Dynamics of engagement in co-creation

Co-creation engagement is pivotal in value co-creation dynamics, as heightened engagement levels are a natural outcome of increased involvement in co-creation processes. The principles of SD logic (Vargo and Lusch, 2016), emphasizing mutual collaboration in value realization, underscore the importance of such engagement. However, our findings indicate that while co-creation engagement is crucial, it does not significantly impact value co-creation behavior on its own. This suggests that engagement alone is insufficient to drive value outcomes, aligning with the expectation-confirmation theory (Oliver, 2010). This theory posits that experiences and expectations must align consistently over time. If subsequent interactions fail to meet these heightened expectations, even highly engaged customers may not exhibit behaviors that enhance value. Interestingly, when co-creation experience and engagement are jointly considered, engagement significantly mediates, albeit negatively, the relationship between co-creation experience and value co-creation behavior. This can be logically explained by the realization that as customers engage more deeply, they may develop higher expectations. If subsequent experiences do not meet these heightened expectations, the perceived value of engaging in co-creation may diminish. This suggests that the interplay between engagement and experience is crucial; positive initial experiences must be consistently maintained to prevent disillusionment.

For academia, the findings prompt a re-evaluation of how scholars conceptualize the role of engagement in value co-creation. Engagement should not be seen as an end in itself but as a facilitator whose impact is influenced by experience. The expectation-confirmation theory's applicability in this context warrants deeper exploration (Oliver, 2010), particularly in understanding potential tipping points or thresholds after which heightened engagement might not yield anticipated value outcomes. This underscores the need to study engagement as a dynamic, evolving construct, influenced by a continuum of experiences rather than a static point of measurement (Lim *et al.*, 2022, 2023).

For industry, understanding the multifaceted nature of co-creation engagement is crucial. While fostering high engagement is undoubtedly beneficial, it is essential to ensure that the subsequent service delivery aligns with the expectations set during initial co-creation experiences. This calls for firms to maintain a consistent quality of interaction and to be attuned to evolving customer expectations. Monitoring tools and feedback mechanisms that capture real-time insights during various stages of the customer journey become invaluable. It is also essential for firms to foster an adaptive mindset, where they are poised to recalibrate their offerings based on continuous feedback, ensuring that high engagement is harnessed effectively to co-create tangible value.

For healthcare providers, fostering patient engagement is essential, but it must be coupled with consistent, high-quality interactions throughout the patient's journey. For example, implementing continuous patient education and follow-up programs can ensure that initial positive engagement is sustained. Health systems can use digital platforms to provide ongoing support and updates, aligning with patients' evolving expectations and needs.

Policymakers can support these initiatives by promoting regulations that ensure healthcare providers maintain high standards of care throughout the patient journey. Policies could incentivize continuous patient engagement and satisfaction metrics, ensuring that healthcare providers are not only engaging patients initially but also maintaining that engagement over time. Additionally, funding for advanced patient management systems that track and respond to patient feedback in real-time could be promoted, ensuring that engagement translates into tangible health outcomes.

### 6.5 Other observations

The complexity of co-creation becomes evident when examining the mediating roles of co-creation self-efficacy and engagement. While traditionally, firms may have prioritized either the service environment or the empowerment of the customer, our findings suggest that both are closely linked. The pathway from co-creation experience to value co-creation behavior can meanders through the terrains of self-efficacy and engagement. This is akin to a journey where the traveler (the customer) is influenced not just by the terrain (the service context) but also by their mindset and preparedness (self-efficacy). Furthermore, the interactional psychology theory emphasizes that behavior is not solely a product of individual traits or situational factors; it emerges from their intersection (Terborg, 1981). In the co-creation context, this implies that the quality of experience, the customer's belief in their capability, and their level of engagement collaboratively sculpt the eventual value co-creation behavior.

Upon detailed scrutiny, the mediation analysis has shone a spotlight on the multifaceted relationships among co-creation experience, co-creation self-efficacy, co-creation engagement, and value co-creation behavior. In particular, the discovery of both positive and negative mediating effects underscores a finer-grained understanding, diverging from linear interpretations and emphasizing the rich complexities within the co-creation process.

The amplifying effect of self-efficacy on the relationship between co-creation experience and engagement resonates with interactional psychology principles. This suggests that behavior in the co-creation context emerges from the confluence of individual traits and the situational environment. When individuals possess strong convictions in their capabilities, it magnifies their level of engagement with the co-creation process, provided they encounter conducive experiential stimuli. This finding aligns with SCT and the work of AbdelAziz *et al.* (2023), who found that the self-efficacy of prosumers impacts their engagement in transforming co-creation intention in fashion products. Similarly, in healthcare, if patients possess co-creation self-efficacy, they are more likely to engage in co-creation of their healthcare services, such as care plans and executions.

Conversely, the dampening effect of both self-efficacy and engagement on the direct pathway from co-creation experience to value co-creation behavior presents an intriguing paradox. One plausible interpretation is that while direct co-creation experiences promote desired behaviors, navigating through self-efficacy and engagement introduces certain complexities. This perspective aligns with the notion that human behaviors, especially in complex scenarios such as co-creation, may not always adhere to predictable or linear trajectories. This signals the potential existence of thresholds of self-efficacy or engagement beyond which their influence becomes counterproductive, opening an exciting avenue for future theoretical exploration.

From a practical standpoint, these findings offer invaluable guidance. The dual nature of the mediators suggests that firms should approach strategy formulation with a sense of balance and caution. For example, simply amplifying co-creation experiences or empowering customers without careful calibration might not lead to linear enhancements in co-creation behavior. It is evident that a harmonious blend is needed. While enhancing the co-creation environment is paramount, there is a parallel need to judiciously foster self-efficacy and engagement. Over-empowering a customer without corresponding support structures, for instance, could lead to a sense of overwhelm, potentially diminishing the overall positive influence on co-creation behavior. Moreover, firms stand to benefit from implementing dynamic feedback loops that not only gauge the end outcome but also monitor the evolving journey of customers. Capturing insights on levels of self-efficacy and engagement can guide firms in fine-tuning their strategies, ensuring that they strike an optimal balance between fostering empowerment, nurturing engagement, and delivering enriching co-creation experiences. This aligns with the SD logic (Vargo and Lusch, 2016), which states that engagement between customers and service providers is paramount, a view supported by Grönroos and Voima (2013). If there is no engagement, there is no co-creation.

For healthcare providers, these findings suggest a finer-grained approach to enhancing patient engagement and self-efficacy. Providers should create supportive environments that facilitate patient involvement without overwhelming them. For instance, chronic disease management programs can integrate step-by-step educational resources that build patient confidence gradually, rather than expecting immediate high engagement. Additionally, healthcare providers can implement personalized coaching sessions that address individual patient needs and self-efficacy levels, ensuring patients feel supported and capable without being overwhelmed.

Policymakers can support these initiatives by developing policies that encourage healthcare providers to adopt balanced engagement strategies. Regulations could mandate regular assessments of patient self-efficacy and engagement, ensuring that healthcare practices are adaptive and responsive to patient feedback. Funding could be allocated to programs that provide continuous training for healthcare professionals on how to foster patient self-efficacy and engagement effectively. Moreover, policies could incentivize healthcare providers to develop and maintain comprehensive support systems that ensure patients remain engaged and empowered throughout their care journey, thereby optimizing health outcomes.

### 6.6 Concluding remarks and future directions

To this end, this has study clarified the interrelationships between co-creation experience, co-creation self-efficacy, co-creation engagement, and value co-creation behavior, offering both scholars and practitioners a deeper understanding of co-creation journeys. As the landscape of service delivery continues to evolve, with customers increasingly positioned as co-creators, it is paramount that we continually refine our understanding of these relationships. Embracing the complexity and interconnectedness of these factors is not just an academic endeavor but also a pragmatic necessity for firms seeking to thrive in an era where value co-creation is becoming the norm. This is particularly true in healthcare, where the integration of co-creation principles can transform patient care. Patients actively participating in their treatment plans can lead to better adherence and outcomes, emphasizing the need for personalized, engaging healthcare experiences. Armed with a finer-grained understanding on how co-creation experiences enhance self-efficacy and engagement, healthcare providers can better develop strategies that not only empower patients but also improve overall care quality.

Through this study, we hope to shed light on some of the paths that lead to successful co-creation, paving the way for future investigations and more informed business strategies, including healthcare entrepreneurship (Lim *et al.*, 2024; Mishra and Pandey, 2023). This study incorporates the co-creation experiences of patients at hospitals, which is a vital part of the healthcare sector. However, co-creation experiences in healthcare may differ from those in other industries such as hospitality and tourism, skill development and training centers, and live streaming marketing platforms. Since healthcare co-creation is highly subjective, the results might not be generalized to every service experience. Therefore, future research should focus on customer co-creation experiences in various contexts and platforms.

Furthermore, this study primarily incorporated personal and behavioral factors of SCT, acknowledging that patient participation and their resources are crucial in healthcare value co-creation (Islam *et al.*, 2024). Future research could incorporate environmental factors to gain a deeper understanding of the context. For instance, observational learning and reinforcement learning (Bandura, 1986) could be explicitly explored to see how patients learn and adapt through their interactions and experiences within the healthcare system. Additionally, while this study focused on co-creation self-efficacy and co-creation engagement, future researchers may consider other mediating variables such as consciousness, literacy, and trust. These factors could further elucidate how various dimensions of SCT influence value co-creation in healthcare.

Moreover, this study found that co-creation self-efficacy and engagement had no direct impact on patient value co-creation behavior but did mediate the relationship between co-creation experience and value co-creation behavior in the context of healthcare. Future research should investigate the impact of self-efficacy and engagement on customer behavior in other settings to enrich the existing literature and current findings. In the midst of doing so, future researchers may also wish to investigate the outcomes of value co-creation behavior, which were not captured in this study, for example, wellbeing or welfare alongside willingness to pay, recommend, and revisit.

Last but not least, further analysis of how enhanced co-creation practices might influence public attitudes or contribute to societal well-being could enrich the discussion. For example, co-creation in healthcare could lead to better patient outcomes, while co-creation in environmental initiatives might foster a greater sense of community responsibility toward sustainability. Exploring the role of digital technologies in facilitating co-creation, examining cross-cultural variations in co-creation practices, and investigating the long-term effects of co-creation on business sustainability are valuable areas for future studies. Innovative methodologies, such as longitudinal studies, experimental designs, or mixed-methods approaches, could further contribute to the body of knowledge. Highlighting potential methodological challenges and how future research might address them would be beneficial. In addition, incorporating the findings into business or marketing curricula could enhance practical understanding and application. Developing case studies based on the research, integrating co-creation projects into coursework, or offering specialized workshops on co-creation strategies could be effective ways to disseminate this knowledge. Educating future business leaders on the pecularities of co-creation enables us to ensure that they are well-equipped to implement these strategies in their professional careers, thus fostering a culture of value co-creation in various sectors. Together, addressing these gaps and expanding the scope of investigation should enable future research to significantly contribute to the theoretical and practical understanding of co-creation in diverse service environments, ultimately leading to better service delivery and enhanced customer experiences across various industries.

## Figures and Tables

**Table 1:** Profile of participants

Characteristic	Category	Frequency (*n*)	Percentage (%)
Gender	Female	101	16.8
	Male	499	83.2
Age (years)	18–26	1	0.2
	27–35	256	42.7
	36–53	291	48.5
	45–53	35	5.8
	Above 53	17	2.8
Marital status	Married	195	32.5
	Not married	405	67.5
Education (highest)	Bachelor	85	14.1
	Master	475	79.2
	Doctorate	33	5.5
	Others	7	1.2
Monthly income (Bangladeshi Taka)	21,000 and below	132	22.0
	21,001–40,000	183	30.5
	40,001–60,000	117	19.5
	60,001–80,000	86	14.3
	80,001 and above	82	13.7
Years of receiving healthcare services from hospitals	Below 2	154	25.7
	2–3	181	30.1
	4–5	85	14.2
	6–7	37	6.2
	Above 7	143	23.8
Type of hospital	Government	149	24.9
	Private	378	63.0
	Autonomous	35	5.8
	Others	38	6.3
Occupation	Student	79	13.2
	Teacher	187	31.1
	Business	16	2.7
	Private job	223	37.1
	Government Job	67	11.2
	Self-employed	28	4.7

**Note(s):** USD1 = ±117 Bangladeshi Taka as of May 20, 2024

**Source(s):** Authors' own compilation

**Table 2:** Confirmatory factor analysis

Construct and item		Convergent validity		Internal consistency or reliability	
		Factor loading	Average variance extracted	Cronbach's alpha	Composite reliability
*Co-creation experience: personalization (PN)*			0.686	0.867	0.867
PN1	I co-create healthcare services	0.832			
PN2	I customize healthcare services as per my requirements	0.834			
PN3	I obtain patient-centric healthcare services through co-creation	0.820			
*Co-creation experience: relative advantage (RA)*			0.561	0.860	0.864
RA1	Co-creation enables me to receive better healthcare services	0.690			
RA2	Co-creation enables me to receive more creative healthcare services	0.834			
RA3	Co-creation enables me to receive more flexible healthcare services	0.833			
RA4	Co-creation enables me to receive higher quality healthcare services	0.750			
RA5	Co-creation enables me to receive healthcare services that better improves my health	0.634			
*Co-creation engagement (EN)*			0.535	0.818	0.820
EN1	I am attracted to co-creation in healthcare services	0.663			
EN2	I learn about co-creation in healthcare services	0.664			
EN3	I pay attention to co-creation in healthcare services	0.824			
EN4	I spend time on co-creation in healthcare services	0.774			
*Co-creation self-efficacy (SE)*			0.602	0.850	0.857
SE1	I know how to manage my health through co-creation of healthcare services	0.762			
SE2	I know the methods to prevent ill health through co-creation of healthcare services	0.853			
SE3	I know how to self-care through co-creation of healthcare services	0.824			
SE4	I know how to access health information through co-creation of healthcare services	0.664			
*Value co-creation behavior: participation behavior (PB)*			0.726	0.939	0.940
PB4	I actively communicate my needs and preferences to the healthcare professionals	0.714			
PB8	I collaboratively engage with healthcare professionals to ensure my health concerns were addressed	0.854			
PB9	I proactively share relevant information with the healthcare professionals to assist in my care	0.894			
PB10	I constructively participate in discussions with healthcare professionals regarding my treatment	0.903			
PB11	I consistently show respect and understanding during interactions with healthcare professionals	0.904			
PB12	I consciously refrain from displaying any negative or aggressive behavior towards healthcare professionals	0.842			
*Value co-creation behavior: citizenship behavior (CB)*			0.631	0.871	0.872
CB1	I speak positively about co-creation of healthcare services to others	0.773			
CB2	I recommend co-creation of healthcare services to my acquaintances	0.853			
CB4	I offer assistance to other patients when they sought to co-create healthcare services	0.783			
CB7	I encourage other patients to co-create healthcare services	0.773			
*Second-order construct*					*Model fit*
Co-creation experience			0.551	0.709	*χ^2^*/df = 2.659, GFI = 0.939, AGFI = 0.920, CFI = 0.969, IFI = 0.969, NFI = 0.951, TLI = 0.964, RMSEA = 0.053
Personalization		0.614			
Relative advantage		0.544			
Value co-creation behavior			0.696	0.887	
Participation behavior		0.900			
Citizenship behavior		0.764			

**Note(s):** Nine items (six items from “value co-creation behavior: participation behavior” and three items from “value co-creation behavior: citizenship behavior”) with factor loadings below 0.70 were removed

**Source(s):** Authors' own compilation

**Table 3:** Correlation matrix and related statistics

	AVE	CR	MSV	MaxR(H)	PN	RA	EN	SE	CB	PB
PN	0.686	0.867	0.195	0.868	*0.828*					
RA	0.561	0.864	0.143	0.879	0.328	*0.749*				
EN	0.535	0.820	0.158	0.836	0.141	0.188	*0.731*			
SE	0.602	0.857	0.158	0.871	0.085	0.113	0.398	*0.776*		
CB	0.631	0.872	0.238	0.877	0.277	0.378	0.145	0.067	*0.794*	
PB	0.726	0.940	0.238	0.949	0.442	0.316	0.109	0.114	0.488	*0.852*
Tolerance					0.799	0.851	0.859	0.873	0.857	0.796
VIF					1.251	1.175	1.164	1.145	1.168	1.256

**Note(s):** Italic diagonals are the square root of average variance extracted (AVEs). CR = compositive reliability. MSV = maximum shared variance. MaxR(H) = maximum H reliability. PN = co-creation experience personalization. RA = co-creation experience relative advantage. EN = co-creation engagement. SE = co-creation self-efficacy. CB = value co-creation citizenship behavior. PB = value co-creation participation behavior. VIF = variance inflation factor

**Source(s):** Authors' own compilation

**Table 4:** Direct relationship

Hypothesis	Relationship	*β*	b	Standard error (S.E.)	Critical ratio (C.R.)	*p*-value	Outcome
H1	Co-creation experience → Value co-creation behavior	0.901	1.132	0.180	6.300	0.000	Supported
H2	Co-creation experience → Co-creation self-efficacy	0.170	0.237	0.095	2.482	0.013	Supported
H3	Co-creation self-efficacy → Value co-creation behavior	0.019	0.017	0.060	0.291	0.771	Not supported
H4	Co-creation experience → Co-creation engagement	0.223	0.252	0.080	3.165	0.002	Supported
H5	Co-creation engagement → Value co-creation behavior	−0.094	−0.104	0.084	−1.247	0.212	Not supported
H6	Co-creation self-efficacy → Co-creation engagement	0.360	0.292	0.042	7.030	0.000	Supported

**Note(s):** C.R. = critical ratio. b represents regression weight and *β* represents standardized regression weight, wherein the standard error, critical ratio, and *p*-value are generated for the former rather than the latter, as per AMOS *v*.24 software output

**Source(s):** Authors' own compilation

**Table 5:** Mediation relationship

Hypothesis	Relationship	Direct effect	*p*-value	Indirect effect	*p*-value	Mediation
H7	Co-creation experience → Co-creation self-efficacy → Value co-creation behavior	0.901	0.000	−1.088	0.027	Competitive (partial) mediation
H8	Co-creation experience → Co-creation engagement → Value co-creation behavior	0.901	0.000	−2.645	0.004	Competitive (partial) mediation
H9	Co-creation experience → Co-creation self-efficacy → Co-creation engagement	0.223	0.002	0.089	0.003	Complementary (partial) mediation

**Source(s):** Authors' own compilation
